# FUTURE DIRECTIONS: DISSEMINATION OF STANDARDIZED GERIATRIC SURGERY CARE NATIONALLY

**DOI:** 10.1093/geroni/igz038.1489

**Published:** 2019-11-08

**Authors:** Mark Katlic

**Affiliations:** Sinai Medical Center, Baltimore, Maryland, United States

## Abstract

The American College of Surgeon’s Coalition for Quality in Geriatric Surgery will formally launch a national initiative aimed to improve the quality of surgical care for all older adults in July 2019. The first-year goal will be to recruit and successfully verify 100+ medical centers. This presentation will provide an overview of dissemination efforts for the standards set for providing high quality surgical care for older adults as well as processes to measure the quality of care provided to older adults at these medical centers. It our vision that this national initiative will lead the effort to the improvement of surgical care of all older adults.

